# ADMET profiling and molecular docking of potential antimicrobial peptides previously isolated from African catfish, *Clarias gariepinus*

**DOI:** 10.3389/fmolb.2022.1039286

**Published:** 2022-12-08

**Authors:** Hedmon Okella, Emmanuel Okello, Andrew Glory Mtewa, Hilda Ikiriza, Bruhan Kaggwa, Jacqueline Aber, Christian Ndekezi, Joseph Nkamwesiga, Clement Olusoji Ajayi, Ivan Mulongo Mugeni, Geofrey Ssentamu, Sylvester Ochwo, Steven Odongo, Casim Umba Tolo, Charles Drago Kato, Patrick Ogwang Engeu

**Affiliations:** ^1^ Veterinary Medicine Teaching and Research Center, School of Veterinary Medicine, University of California, Davis, Tulare, CA, United States; ^2^ Pharm-Biotechnology and Traditional Medicine Centre, Mbarara University of Science and Technology, Mbarara, Uganda; ^3^ Department of Population Health and Reproduction, School of Veterinary Medicine, University of California, Davis, Davis, CA, United States; ^4^ Chemistry Section, Malawi Institute of Technology, Malawi University of Science and Technology, Limbe, Malawi; ^5^ Department of Pharmacy, College of Health Sciences, Makerere University, Kampala, Uganda; ^6^ Department of Pharmacy, Faculty of Medicine, Gulu University, Gulu, Uganda; ^7^ MRC/UVRI and LSTMH Uganda Research Unit, Entebbe, Uganda; ^8^ International Livestock Research Institute, Nairobi, Kenya; ^9^ Institut für Virologie, Freie Universität, Berlin, Germany; ^10^ Medical Entomology Laboratory, Infectious Diseases Research Collaboration, Kampala, Uganda; ^11^ Department of Biotechnical and Diagnostic Sciences, College of Veterinary Medicine, Animal Resources and Biosecurity, Makerere University, Kampala, Uganda; ^12^ Center for Animal Health and Food Safety, University of Minnesota, St. Paul, MN, United States

**Keywords:** ADMET profiling, African catfish, antimicrobial peptides, novel leads, molecular docking

## Abstract

Amidst rising cases of antimicrobial resistance, antimicrobial peptides (AMPs) are regarded as a promising alternative to traditional antibiotics. Even so, poor pharmacokinetic profiles of certain AMPs impede their utility necessitating, a careful assessment of potential AMPs’ absorption, distribution, metabolism, excretion, and toxicity (ADMET) properties during novel lead exploration. Accordingly, the present study utilized ADMET scores to profile seven previously isolated African catfish antimicrobial peptides (ACAPs). After profiling, the peptides were docked against approved bacterial protein targets to gain insight into their possible mode of action. Promising ACAPs were then chemically synthesized, and their antibacterial activity was validated *in vitro* utilizing the broth dilution method. All seven examined antimicrobial peptides passed the ADMET screening, with two (ACAP-IV and ACAP-V) exhibiting the best ADMET profile scores. The ACAP-V had a higher average binding energy (−8.47 kcal/mol) and average global energy (−70.78 kcal/mol) compared to ACAP-IV (−7.60 kcal/mol and −57.53 kcal/mol), with the potential to penetrate and disrupt bacterial cell membrane (PDB Id: 2w6d). Conversely, ACAP-IV peptide had higher antibacterial activity against *E. coli* and *S. aureus* (Minimum Inhibitory Concentration, 520.7 ± 104.3 μg/ml and 1666.7 ± 416.7 μg/ml, respectively) compared to ACAP-V. Collectively, the two antimicrobial peptides (ACAP-IV and ACAP-V) are potential novel leads for the food, cosmetic and pharmaceutical industries. Future research is recommended to optimize the expression of such peptides in biological systems for extended evaluation.

## Introduction

Antimicrobial peptides (AMPs) are short, amphipathic gene-encoded endogenous peptides, largely with a net positive charge (Hancock and Sahl, 2006; Fjell et al., 2012). They are evolved in all forms of life as effector molecules of the innate immune system, guarding the host against microbial invasion and hence the alternative name Host Defense Peptides (HDP) (Ravichandran et al., 2010). They are quick-acting (Raheem and Straus, 2019), short in length, and the majority are Generally Recognized As Safe (GRAS) (Hansen et al., 2020). Interestingly, AMPs have the potential to target both the intracellular and membrane (lytic) protein receptors (Hancock, 2001; Wilcox, 2004; Brogden, 2005). Moreso, AMPs have been reported to have a low risk of pathogen resistance. Accordingly, peptides and proteins are now gradually gaining ground over small molecule-based drug searches (Otvos and Wade, 2014). For example, up to 239 peptides and proteins have been approved by US Food and Drug Authority (FDA) for clinical use (Usmani et al., 2017), accounting for 3.5% of the FDA-approved drugs (Wishart et al., 2018). Despite the optimistic trend and potential of AMPs as a promising alternative to traditional antibiotics, poor pharmacokinetics profiles (Hou and Wang, 2008; Cumming et al., 2013) are still among the major carryon defect of most novel antimicrobial peptide candidates. One probable approach to address such an impediment is the deployment of machine learning techniques for virtual screening of antimicrobial peptide libraries based on Absorption, Distribution, Metabolism, Excretion and Toxicity (ADMET) scores, at the basic stages of any potential drug candidate search (Hou and Wang, 2008; Cumming et al., 2013).

Considering the above, the current study profiled and evaluated the *in vitro* activity of candidate AMPs derived from the African catfish, *Clarias gariepinus* (Burchell, 1822). The African catfish is a scale-less bottom dweller in the calm pathogen-dense freshwaters (Aliyu and Solomon, 2012), and produces extra mucus compared to scaled fish for a more effective innate immune response (Dash et al., 2018). Their skin mucus is composed of antimicrobial peptides, flavoenzymes, clotting factors, complement proteins, hemolysins, immunoglobulins, glycoproteins, and hydrolytic enzymes responsible for inhibiting microbial activity as well as immune modulation (Välimaa et al., 2019). Fascinatingly, Reverter and colleagues contend that of these various fish skin mucus components, antimicrobial peptides are the most promising strategy to inhibit bacteria in the era of antimicrobial drug resistance (Reverter et al., 2018). The GRAS status of AMPs, their short sequences and rapid onset of action have been fronted as AMPs’ stronghold (Aoki and Ueda, 2013; Guilhelmelli et al., 2013). In our previous study, seven African Catfish Antimicrobial Peptides (ACAPs) were identified for the first time (Okella et al., 2021). However, the investigation did not attempt to assess the ADMET profiles of such AMPs. Besides, the possible mode of action and the *in vitro* activity of the most promising AMPs from such libraries were not explored. Thus, to contribute to potential drug leads in this era of a new antimicrobial drug search, we present findings of the ADMET profiling and early validation of promising AMPs previously isolated from the skin mucus of African catfish.

## Materials and methods

### Study design

An experimental study design was adopted; Both *in silico* and *in vitro* approaches were employed to collect the data. Seven previously identified antimicrobial peptides from African catfish, *Clarias gariepinus* skin mucus (Okella et al., 2021), were studied.

### Absorption, distribution, metabolism, excretion, and toxicity screening of antimicrobial peptides

The Simplified Molecular Input Line Entry System (SMILES) structural format of the seven previously identified AMPs was obtained using a web-based tool, PepSMI (https://www.novoprolabs.com/tools/convert-peptide-to-smiles-string). PepSMI runs an algorithm that converts raw sequence files into a string of texts, unambiguously describing each atom and bond in the molecule in a manner amenable to machine processing. Thereafter, Absorption, Distribution, Metabolism, Excretion and Toxicity (ADMET) profiles involving key parameters like Human Intestinal Absorption (HIA), mutagenicity, carcinogenicity, central nervous system (CNS) permeability, Drug-Induced Liver Injury (DILI), cytochrome P450 enzymes inhibition, clearance, Half-life, and Skin sensitization were assessed using the latest version (July 2021 Release) of ADMETlab 2.0 platform (https://admetmesh.scbdd.com/). As previously described (Xiong et al., 2021), the SMILES files of the AMPs were submitted to the platform. Analysis was executed based on a comprehensive database composed of 0.25 million entries from PubChem, Online chemical modeling environment (OCHEM), Drug-Bank, Chemical Database at European Molecular Biology Laboratory (ChEMBL), Toxicity Estimation Software Tools (TEST) developed by the U.S. Environmental Protection Agency (EPA) and peer-reviewed literature (Xiong et al., 2021). Thereafter, the platform profiled AMPs based on their respective ADMET scores. Pan Assay Interference Compounds (PAINS) and undesirable reactive compounds were then filtered from Screening Libraries by implementing the PAINS rule (Baell and Holloway, 2010) and Bristol-Myers Squibb (BMS) rules (Pearce et al., 2006) respectively. Furthermore, Lipinski’s “Rule of 5” (RO5) were employed to assess the drug-likeness properties of the antimicrobial peptides. The pharmacokinetic parameter predictions were based on the corresponding basic information and experimental values of the respective entries at the platform. Overall, the AMPs with the best ADMET profiles were then, prepared for the molecular docking exercises.

### Ligand preparation

Ligands were prepared as previously described (Okella et al., 2020). Briefly, antimicrobial peptide FASTA file sequences were inputted to predict the 3-D structure of the AMPs utilizing the Iterative Threading Assembly Refinement (ITASSER) server (https://zhanglab.dcmb.med.umich.edu/I-TASSER/) (Yang and Zhang, 2015). The server utilizes protein templates identified by the Local Meta-Threading Server (LOMETS) from the Protein Data Bank (PDB) library (https://www.rcsb.org/) (Berman et al., 2002). LOMETS uses multiple threading approaches to align the query protein amino acid sequence against the PDB. Template proteins with the highest sequence identity and z-score were used in the modeling exercise (Supplementary Material S1). The best models were identified using a −5 to 2 confidence score (c-score) scale, with a higher c-score value signifying models with a high confidence score and *vice versa*. The c-score is calculated based on the significance of threading template alignments and the convergence parameters of the structure assembly simulations (decoys). The predicted 3-D structures of the peptides were cross-validated using a web-based *de novo* peptide structure prediction tool, PEP-FOLD V3.5 at https://bioserv.rpbs.univ-paris-diderot.fr/services/PEP-FOLD/ (Thevenet et al., 2012). In the procedure, FASTA files were inputted, default 100 simulations execute, and the best output models were ranked based on the coarse-grained optimized potential for efficient structure prediction (sOPEP) energies and Apollo predicted melting temperature (t_m_). Models with the lowest sOPEP energies and highest t_m_ were ranked as the best model. Thereafter, a two-phased quality check was performed on best-modeled structures (I-TASSER and PEP-FOLD v3.5 modeled); Initially, a web-based Protein Structure Analysis (ProSA) tool (https://prosa.services.came.sbg.ac.at/prosa.php) (Wiederstein and Sippl, 2007) was used. The ProSA tool predicts the query protein z-score, residual energy, and plots the local model quality. The ProSA z-score equates the query protein z-score against experimentally validated proteins in the PDB library. A higher z-score value indicates greater similarity. Later, the stereo-chemical properties of the modelled antimicrobial peptides in PDB file format were estimated using a Ramachandran Plot Server at https://zlab.umassmed.edu/bu/rama/ (Anderson et al., 2005). Here, the residues’ bonding patterns and their effects on the distribution of backbone dihedral angles were revealed in a Ramachandran Plot (Hollingsworth and Karplus, 2010). Models with majority residues in the most favoured region (>83%) and none or few residues (<3%) in the disallowed region were qualified as the best quality models.

### Target fishing and preparation

Targets were selected based on previous reports on their vital roles in the selected bacteria. The selection utilized DrugProt, an EMBL-EBI web server at https://www.ebi.ac.uk/thornton-srv/databases/drugport/. DrugPort is based on the latest (December 2021) version of the DrugBank database, and it contains 1492 approved drug targets and 1664 unique protein targets. Here, the best ADMET profiled peptide sequences in FASTA file format were submitted as input files. The target, PDB identity, percentage target identity, z-score, and organism’s name were retrieved and used to qualify protein targets in bacteria. Only targets with experimentally determined structures in the PDB and known function were considered. Thereafter, the 3-D structures and class of the receptor proteins were retrieved from the PDB library based on X-ray crystallography (XRC) and Nuclear Magnetic Resonance (NMR) data. In addition, the lytic mode of action of the promising antimicrobial peptides was investigated by empirically retrieving the bacterial cell membrane protein (PDB Id: 2w6d). All the retrieved 3-D target protein structures were visually inspected using PyMOL software. The BOVIA Discovery Studio v2021 client was used to clean the 3-D structures of protein receptors and define the attributes of the binding site. During cleaning, water molecules, ligand groups and heteroatoms were dispelled from the receptor proteins before the docking exercise to ensure energy minimization for a more factual docking output. Defining the binding site attributes, on the other hand, involved adding the hydrogen before utilization of the receptor cavity method to unveil the docking box dimensions across the coordinates.

### Molecular docking studies

To gain insight into the possible mechanism of action, AutoDock VINA (Trott and Olson, 2019) at DINC 2.0 Web server (http://dinc.kavrakilab.org/) (Antunes et al., 2017) was used to perform several molecular docking calculations. Here, the PDB file format for both ligand and receptor protein was submitted as input files with the receptor protein cantered in the previously defined docking box. The binding energies (BE, kcal/mol) and Root Mean Square Deviation (RMSD, A°) were outputted. Thereafter, PatchDock, an online docking server (https://bioinfo3-D.cs.tau.ac.il/PatchDock/php.php) was utilized in a cross-validation exercise. PatchDock’s molecular docking algorithm is based on Shape Complementarity Principles (Duhovny et al., 2002; Schneidman-Duhovny et al., 2005). Here, ligand and receptor PDB files were uploaded to the server and later run at a default complex type and maximum Root Mean Square Deviation (RMSD) of 4.0. The output transformation files (best 1000 transformations) were then refined and rescored using Fast Interactive Refinement in molecular Docking (FireDock) (https://bioinfo3-D.cs.tau.ac.il/FireDock/php.php) to generate global energies of the docked complexes. Global energy is generated by comparing the low-energy minima with the experimental values (Mashiach et al., 2008). The PDB structure of the complex with the highest binding energy was retrieved. Myxinidin was the control drug in the docking exercise for comparison with the most promising peptide ligand. All ligand-receptor complexes were rendered in BOVIA Discovery Studio v2021 software and Chimera v1.15 (Pettersen et al., 2004), during which the structural parts that are important to the biological activity of the two promising leads were identified.

### Peptide synthesis

**FIGURE 1:**
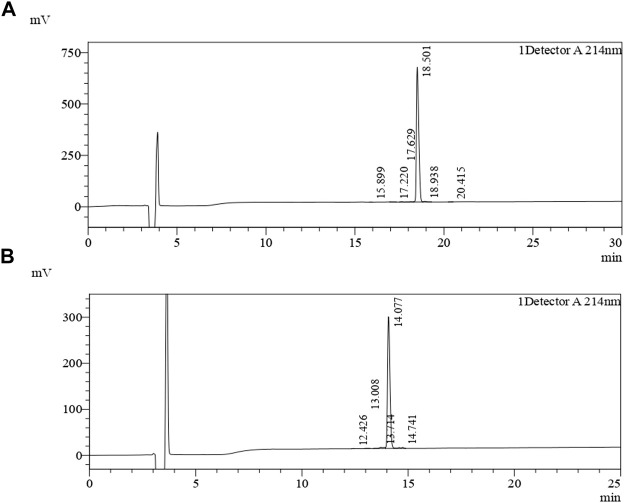
Chromatograms of the purified African catfish antimicrobial peptides. The synthesized peptides were loaded on a YMC-Triart C18 (4.6 × 250 mm × 5 um) reversed-phase highperformance liquid chromatography (RP-HPLC) column equilibrated with 0.1% (v/v) trifluoroacetic acid (TFA)/water. Gradient elution was carried out with 0.1% (v/v) acetonitrile/TFA at a flow rate of 1.0 ml/min. **(A)** Chromatogram for ACAP-IV in the 30 min run at 214 nm. **(B)** Chromatogram for ACAP-V in the 25 min run at 214 nm. In both cases, 25 µl of samples were injected utilizing 100% water dissolution method.

**FIGURE 2:**
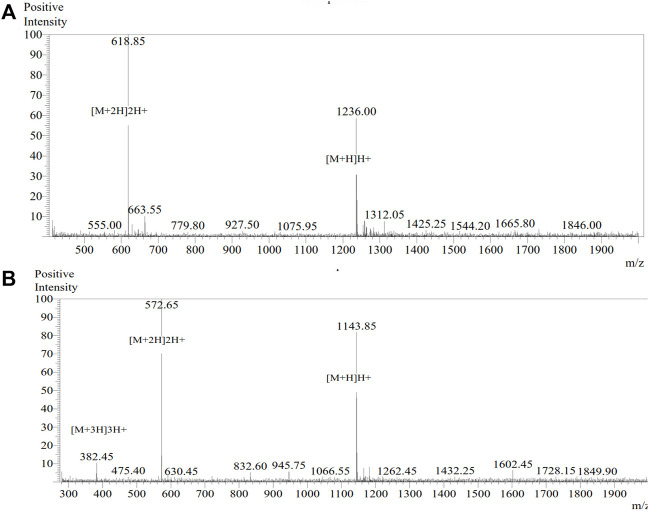
MS spectra for the African catfish antimicrobial peptides. Here, 15% ACN plus 85% water dissolution method was used prior to 0.2 µl injection. Electronspray Ionization (ESI) interface was used with the detector set at −2.4 kV. **(A)** MS spectrum of peptide ACAP-IV (KVSKVLHKAIL) with a theoretical mass of 1235.57 and an observed mass of 1235.70. **(B)** MS spectrum of peptide ACAP-V (VVLGSGGVGKSAL) with a theoretical mass of 1143.34 and an observed mass of 1143.30.

Of the seven, two promising African catfish antimicrobial peptides (ACAP-IV and ACAP-V) were chemically synthesized by Biomatik Cooperation (Wilmington, United States). The synthesis process involved a condensation reaction between the carboxylic terminus and amino terminus to join the amino acid residues through peptide bonds (Marglin and Merrifield, 1970). Unlike in protein biosynthesis, in which the amino terminus is coupled to the carboxylic terminus, here the synthesis is achieved by coupling of the carboxylic group of the incoming amino acid to the amino terminus. The synthesized peptides were then purified by preparative High-Performance Liquid Chromatography (HPLC) using a YMC-Triart C_18_ analytic column (4.6 × 25 mm × 5 um). The linear gradient used was from solvent A (0.1% Trifluoroaceticacid in 100% Water) to solvent B (0.1% Trifluoroacetic in 100% Acetonitrile). A flow rate of 1.0 ml/min and run-time of 30 min. The peptides were detected at 214 nm using UV spectrophotometry (Figure 1), with a purity of 99.01% and 96.48% for ACAP-IV and ACAP-V, respectively. The molecular masses were confirmed by mass spectrometry (Figure 2). Synthesized peptides were stored at −20°C and resuspended in distilled, deionized water following the manufacturer’s recommendation.

### Antibacterial properties of the synthesized African catfish antimicrobial peptides

The broth dilution method, as previously described by Teh et al. (2017), was utilized with minor modifications using *Staphylococcus aureus* (ATCC 25923) and *Escherichia coli* (ATCC 25922) as test organisms. Briefly, a two-fold dilution of each synthesized protein (ACAP-IV and ACAP-V) was made by adding 100 μl of the concentrated peptide solution (10 mg/ml) to an equal volume of Tryptic Soy Broth (TSB) in each well of a 96-well cell culture plate (Dynatec, El Paso, United States). Subsequently, a two-fold serial dilution was performed by transferring 100 μl of the mixture to an equal volume of TSB in the next well, up to the 8th dilution. Lastly, 100 μl of the mixture was discarded from the eighth well. Similarly, two-fold serial dilutions of the positive control, 50 μg/ml ciprofloxacin (Cyman, Ann Arbor, United States) and the negative controls (distilled deionized water and TSB) in TSB were made. Thereafter, 5 μl of diluted bacterial suspension (1.5 × 10^6^ cells/ml) was added to all test wells and mixed thoroughly. Micro-dilution was performed in triplicates for each bacterial species. After overnight incubation at 37°C, 5 μl of 6.75 mg/ml Resazurin (Thermo Scientific, Ward Hill, United States) was added to all wells and incubated at 37°C for another 4 h. Color changes were physically observed and recorded, with microbial growth implied by an irreversible colour change from the blue resazurin to pink resorufin. The lowest concentration without colour change was considered the Minimum Inhibitory Concentration (MIC).

## Results

### Absorption, distribution, metabolism, excretion, and toxicity screening of antimicrobial peptides

Examining the pharmacokinetic attributes of a lead molecule is vital in assessing its efficacy and indemnity. In the present study, various parameters, including Human Intestinal Absorption (HIA), mutagenicity, carcinogenicity, Central Nervous System (CNS) permeability, drug induced liver injury, and Cytochrome P450 enzymes inhibition among others were examined. When administered orally, the HIA value is a key parameter that influences bioavailability. Just like Myxinidin, the seven peptides were Human Intestinal Absorption positive (HIA+), with an HIA score above 0.1, thus can easily be absorbed in the human intestine (Table 1). The ability of a compound to cross the blood-brain barrier, on the other hand, is determined by Blood-Brain Barrier (BBB) permeability index. Compounds with a BBB value greater than or equal to 0.3 can cross the BBB and potentially cause CNS toxicity. All the seven antimicrobial peptides evaluated, just like Myxinidin, were unable to cross the BBB (BBB score <0.1, BBB-), under optimal distribution volume (0.04–20 L/kg). Further, none of the compounds was found to inhibit any of the cytochrome P450 enzymes (CYP1A2 and CYP3A4). Much like Myxinidin, all peptides studied demonstrated low clearance and short half-life. In addition, they were found to be non-blockers of the Human ether ago-go gene (hERG) (hERG score <0.5), non-carcinogenic, non-mutagenic and non-skin sensitive in nature. Neither Pan Assay Interference Compounds (PAINS) nor Bristol-Myers Squibb’s (BMS) undesirable reaction alerts were detected during the screening process. Therefore, based on the above parameters, the AMPs considered in this study were predicted safe and reliable, with ACAP-IV and ACAP-V demonstrating the best ADMET profiles. However, given the high molecular weight of the 7 ACAPs (>500 Da), all the ACAPs violated the Lipinski’s Rule of 5 (Table 2), suggesting their poor absorption when orally administered.

**TABLE 1:** Predicted ADMET scores of the antimicrobial peptides.

Category	Property (unit)	ACAP-IV	ACAP-V	ACAP-II	ACAP-VI	ACAP-VII	ACAP-I	ACAP-III	Myxinidin	Inference/references range
Absorption	HIA/Human Intestinal Absorption (%)	0.959	0.977	0.981	0.984	0.986	0.992	0.995	0.997	HIA>0.3: HIA Positive, HIA<0.3: HIA Negative
	Papp/Caco-2 permeability (cm/s)	−6.879	−8.103	−7.431	−7.535	−6.790	−7.479	−8.372	−7.629	Optimal: Higher than -5.15
Distribution	VD/Volume Distribution (L/Kg)	0.480	0.431	0.478	0.426	0.359	0.450	0.370	0.601	Optimal: 0.04–20L/Kg
	BBB/Blood brain barrier penetration (%)	0.024	0.048	0.041	0.055	0.014	0.042	0.042	0.036	BBB≥0.1: BBB Positive, BBB<0.1: BBB Negative
	PPB/Plasma protein binding (%)	18.546	9.850	15.764	13.936	25.372	16.507	19.747	22.250	PPB<90%: Optimal; PPB>90%: Low Therapeutic Index
Metabolism	CYP1A2-Inhibitor	0.000	0.000	0.000	0.000	0.000	0.000	0.000	0.000	>0.5: An inhibitor; <0.5: Non inhibitor
	CYP1A2-Substrate	0.002	0.000	0.000	0.001	0.000	0.000	0.000	0.002	>0.5: A substrate; <0.5: Non substrate
	CYP3A4-Inhibitor	0.056	0.004	0.011	0.016	0.003	0.009	0.001	0.025	>0.5: An inhibitor; <0.5: Non inhibitor
	CYP3A4-Substrate	0.006	0.001	0.000	0.003	0.000	0.000	0.000	0.000	>0.5: A substrate; <0.5: Non substrate
Excretion	CL/Clearance (ml/min/Kg)	1.584	1.059	1.004	1.319	1.066	0.925	0.372	1.256	High: >15 ml/min/kg; Moderate: 5–15 ml/min/kg; Low: <5 ml/min/kg
	T_1/2_/Half-life (H)	0.934	0.830	0.869	0.828	0.742	0.870	0.876	0	Long half-life: >3 h; Short half-life: <3 h
Toxicity	hERG blockers	0.010	0.001	0.003	0.007	0.005	0.003	0.000	0.005	>0.5: Blocker; <0.5: Non blocker
	DILI/Drug Induced Liver Injury	0.009	0.002	0.001	0.002	0.000	0.001	0.005	0.006	>0.5: Toxic to liver; <0.5: Non-toxic to liver
	AMES (Ames mutagenicity)	0.007	0.004	0.005	0.003	0.002	0.004	0.003	0.003	>0.5: Positive; <0.5: Negative
	Carcinogenicity	0.011	0.005	0.012	0.002	0.030	0.011	0.001	0.062	>0.5: Carcinogen; <0.5: Non Carcinogen
	Skin sensitization	0.104	0.236	0.230	0.093	0.098	0.105	0.299	0.186	>0.5: Sensitizer; <0.5: Non sensitizer

ACAP, African catfish antimicrobial peptides; hERG, Human ether ago-go gene; CYP, cytochrome P450.

**TABLE 2:** The seven African Catfish Antimicrobial Peptides used in the study.

Seq. ID	Sequences	Length	Molecular weight	LogP	H_acc_	H_don_
ACAP-I	AALKKALTAGGY	12	1163.68	−1.8	29	20
ACAP-II	AALKKALAAGGY	12	1133.67	−0.7	28	19
ACAP-III	GVASAPASGTGGFSFG	16	1369.64	−3.4	37	21
ACAP-IV	KVSKVLHKAIL	11	1235.82	0.7	29	21
ACAP-V	VVLGSGGVGKSAL	13	1143.67	−1.2	30	19
ACAP-VI	FGGAGVGKTVL	11	1005.57	−0.4	25	16
ACAP-VII	IAIIPSKKLRNKIAG	15	1622.05	0.1	40	26

ACAP, African catfish antimicrobial peptides; Seq. ID, Sequence Identity. LogP, logarithm of the n-octanol/water distribution coefficient; Hacc, number of hydrogen acceptors; Hdon, number of hydrogen donors. Peptide sequences were adopted from (Okella et al., 2021).

### Ligand preparation

**FIGURE 3:**
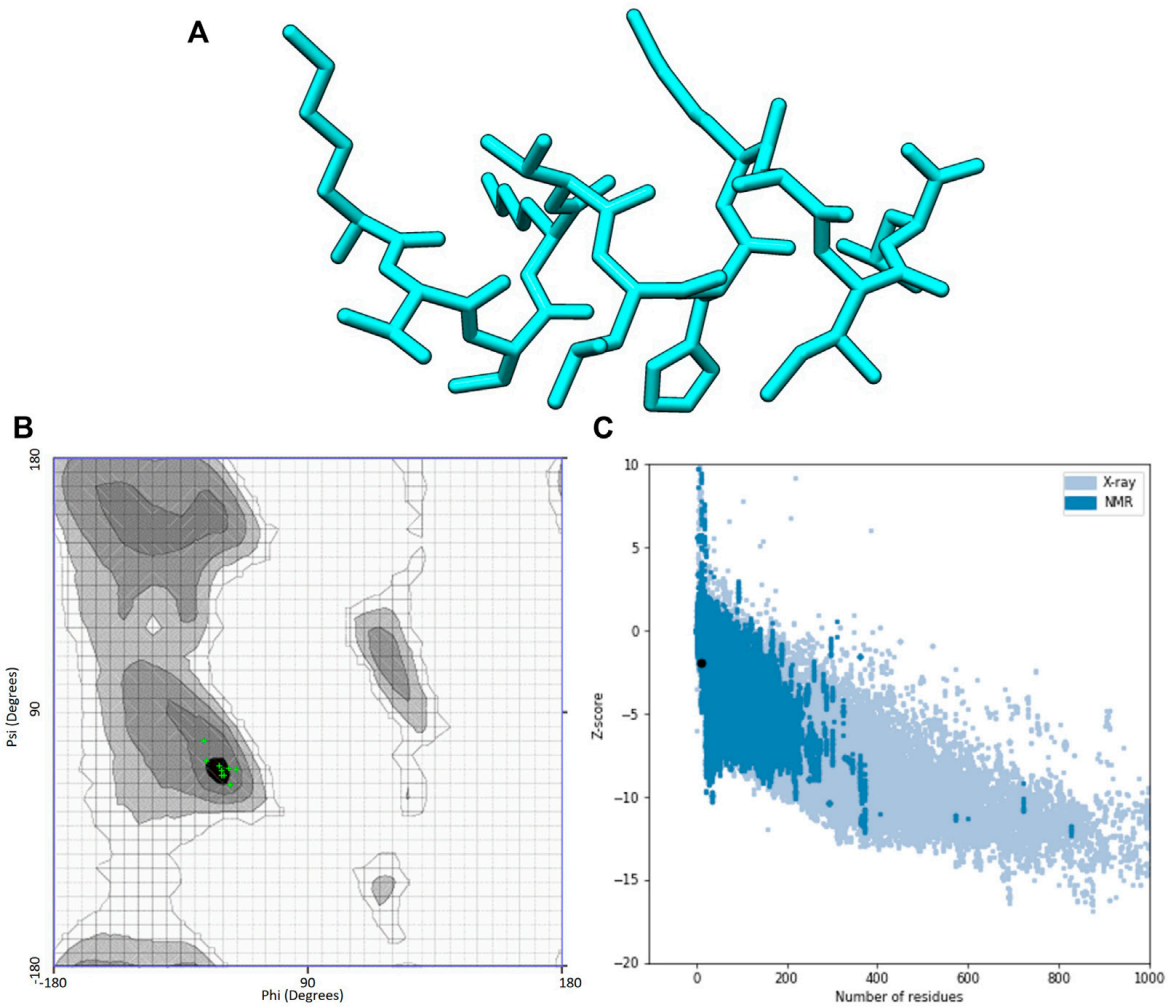
PEP-FOLD modelled peptide 3-D structures of ACAP-IV together with respective Ramchndran and ProSA validation plots. **(A)** Stick format of PEP-FOLD modelled ACAP-IV. **(B)** PEP-FOLD-modelled ACAP-IV Ramachandran plot. **(C)** PEP-FOLD-modelled ACAP-IV ProSA *z*-score. PEP-FOLD-modelled ACAP-I peptide had all (100%) amino acid residues in the most favoured region with a *z*-scores of −1.91.

**FIGURE 4:**
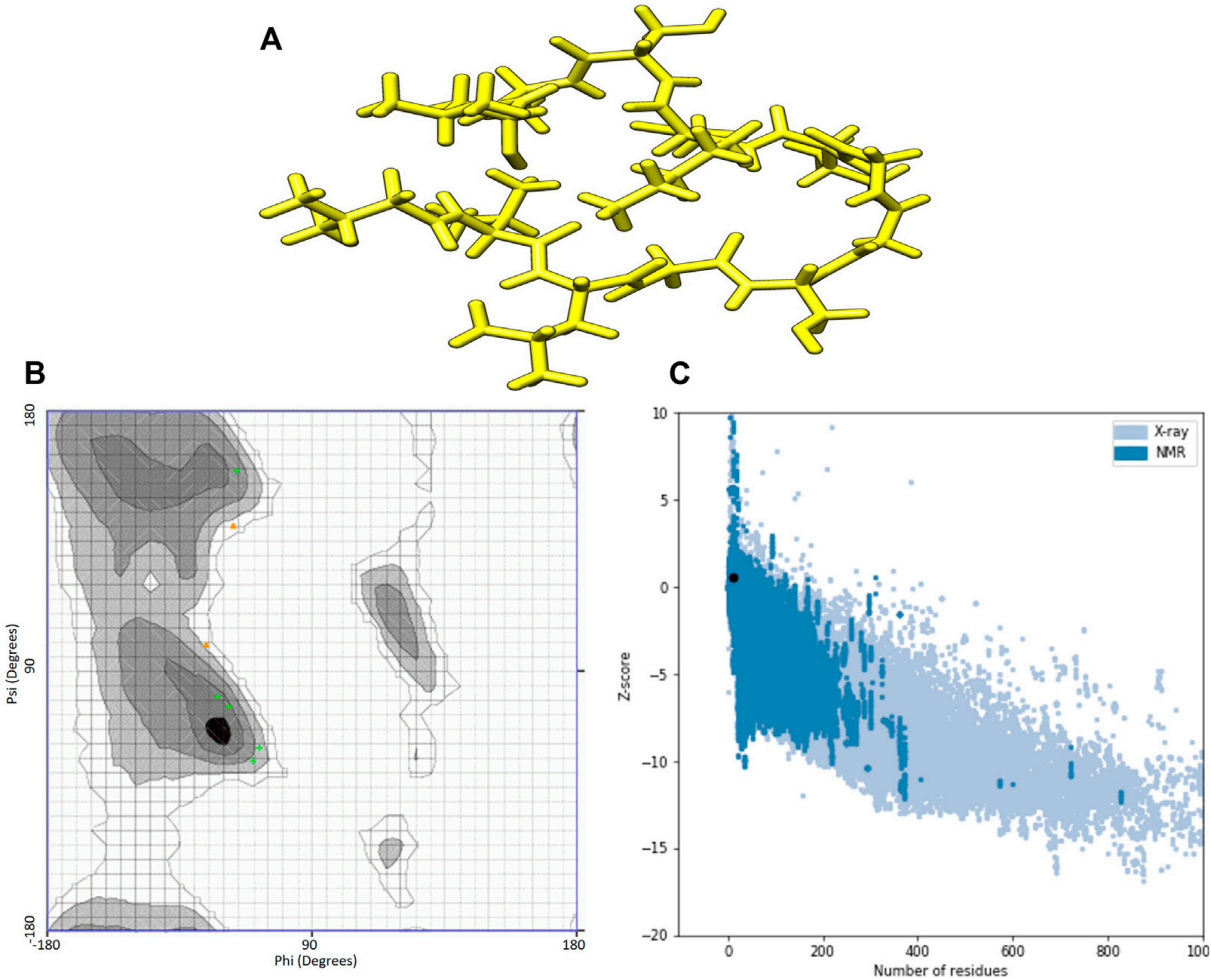
I-TASSER modelled peptide 3-D structures of ACAP-V together with respective Ramchndran and ProSA validation plots. **(A)** Stick format of I-TASSER modelled ACAP-V. **(B)** I-TASSER -modelled ACAP-V Ramachandran plot. **(C)** I-TASSER-modelled ACAP-V ProSA *z*-score. PEP-FOLD-modelled ACAP-I peptide had majority of its residues in the most favoured region (71.5%) amino with the higest highest *z*-scores of 0.59.

I-TASSER predictions outputted two models for ACAP-IV and five for ACAP-V peptides. Guided by the c-scores, models were selected for downstream analysis, with all model_1 in both AMPs having the highest c-score ranking and were thus, considered the best I-TASSER models. On the other hand, maximum numbers of models (five) were observed across all the PEP-FOLD modeled 3-D structures (Table 3). Just like in I-TASSER predictions, all the PEP-FOLD model_1 across the two peptides demonstrated the lowest sOPEP energy with the highest Apollo predicted melting temperature (tm) and were therefore considered the best models for downstream analysis. Upon subjecting the I-TASSER and PEP-FOLD best models to structure quality evaluations, PEP-FOLD models demonstrated a better quality than I-TASSER’s and were considered for the molecular docking exercises. Here, the Ramachandran plot demonstrated that PEP-FOLD modeled structures for ACAP-IV (Figure 3) and ACAP-V peptides had all their residues (100%) in favoured the most favoured region (Table 4), unlike in the ACAP-V I-TASSER modeled 3-D structure with only 71.5% of its residues in the most favoured region and the rest in the additional allowed region (Figure 4). Given that, ProSA yielded analogous z-scores, Ramachandran plots were heavily relied upon.

**TABLE 3:** Top output models with respective scores as predicted by I-TASSER and PEP-FOLD.

Peptide Id	I-TASSER output model *c*-score					PEP-FOLD output model sOPEP and tm scores									
	Model1	Model2	Model3	Model4	Model5	Model1		Model2		Model3		Model4		Model5	
						sOPEP	t_m_	sOPEP	t_m_	sOPEP	t_m_	sOPEP	t_m_	sOPEP	t_m_
ACAP-IV	−0.09	−3.40	—	—	—	−15.82	0.911	−15.75	0.895	−15.73	0.921	−15.72	0.912	−15.69	0.939
ACAP-V	−0.96	−3.33	−1.37	−2.64	−3.04	−8.29	0.379	−8.24	0.323	−7.99	0.353	−7.98	0.289	−7.94	0.359

ACAP, African catfish antimicrobial peptides; t_m_, Apollo melting temperature; *c*-score, confidence score.

**TABLE 4:** Evaluation scores for the predicted 3-D structures of African catfish antimicrobial peptides.

Tool	Parameters	Models	ACAP-IV	ACAP-V
Ramachandran Plot	Residues in most favoured regions (%)	I-TASSER	100.0	71.5
		PEP-FOLD	100.0	100.0
	Residues in additional allowed regions (%)	I-TASSER	0.0	28.5
		PEP-FOLD	0.0	0.0
	Residues in generously allowed regions (%)	I-TASSER	0.0	0.0
		PEP-FOLD	0.0	0.0
	Residues in disallowed regions (%)	I-TASSER	0.0	0.0
		PEP-FOLD	0.0	0.0
ProSA	*z*-score	I-TASSER	−2.05	0.59
		PEP-FOLD	−1.91	0.28

ProSA, protein structure analysis; ACAP, African catfish antimicrobial peptides.

### Target fishing and preparation

A total of 11 drug targets were retrieved from the DrugBank database (Supplementary Material S2). Most of the receptor proteins (27.3%) were of *Escherichia coli* origin. Ferredoxin-dependent glutamate synthase 2 (PDB Id: 3ozw), demonstrated the highest target z-score (96.8%), followed by Lipid binding protein Cholesterol oxidase (89.1%) (PDB Id: 2gew). The lowest z-score (77.5%) was observed in Dihydrolipoyl dehydrogenase (PDB ID: 1ojt). All the PDB retrieved 3-D structures were previously elucidated by X-Ray crystallography (Berman et al., 2002).

### Molecular docking studies

To explore the interaction between the protein targets and antimicrobial peptides, molecular docking, a widely adopted and reliable modeling approach (Sulaiman et al., 2019), was performed using AutoDock, PatchDock, and FireDock docking engines. Across the docking engines used, independent docking of individual peptides against respective bacterial target receptors was found to support their possible antibacterial role with average binding energy (BE) and global energy (GE) of −7.80 kcal/mol and −65.24 kcal/mol, respectively. Generally, the peptides were able to bind with relatively high binding energies ranging from −6.10 to −9.40 kcal/mol and global energies of −51.51 to −88.84 kcal/mol (Table 5). The ACAP-V peptide demonstrated higher average binding energy (−8.47 kcal/mol) against respective protein targets compared to ACAP-VI (−7.60 kcal/mol). This was not any different when PatchDock and FireDock were used, as ACAP-V peptide demonstrated higher average global energy (−70.78 kcal/mol), unlike ACAP-VI (−57.53 kcal/mol).

**TABLE 5:** Docking energies of the antimicrobial peptides against intracellular targets.

Biological targets	PDB Id	Ligand/PubChem CID	Binding energy (kcal/mol)	PatchDock solution #	Global energy (kcal/mol)	RMSD (Å)
Ferredoxin-dependent glutamate synthase 2	1ofd	ACAP-IV	−7.20	34	−59.96	0.0
		643976	−7.50			0.0
Penicillin-binding protein 2x	1mwt	ACAP-IV	−7.20	421	−53.05	0.0
		5289182	−6.80			0.0
30 S ribosomal protein S13	4v6l	ACAP-IV	−6.90	31	−66.16	0.0
		32051	−7.00			0.0
Aminoglycoside N (6′)-acetyltransferase type 1	1s3z	ACAP-IV	−7.60	306	−53.51	0.0
		33042	−7.40			0.0
Acyl-homoserine lactone acylase PvdQ	4wks	ACAP-IV	−-6.10	137	−54.99	0.0
		521154	−6.90			0.0
Flavohemoprotein	3ozw	ACAP-V	−9.20	215	−57.19	0.0
		46936853	−9.10			0.0
Thioredoxin reductase	1f6m	ACAP-V	−8.50	536	−51.51	0.0
		445515	−8.70			0.0
NADH peroxidase	1nph	ACAP-V	−8.40	60	−88.84	0.0
		271	−8.70			0.0
Fumarate reductase flavoprotein	1kf6	ACAP-V	−7.00	376	−80.64	0.0
		1561	−5.90			0.0
Monomeric sarcosine oxidase	2gb0	ACAP-V	−9.40	104	−65.45	0.0
		1061	−7.20			0.0
Dihydrolipoyl dehydrogenase	1ojt	ACAP-V	−8.20	91	−81.04	0.0
		643975	−8.40			0.0
Monomeric sarcosine oxidase	2gb0	Myxinidin	−7.80	219	−46.8	0.0
NADH peroxidase	1nph	Myxinidin	−6.60	334	−68.25	0.0

ACAP, African catfish antimicrobial peptides; NADPH, nicotinamide adenine dinucleotide phosphate; FAD, Flavin Adenine Dinucleotide; RMSD, root mean square deviation; Å, Ångström.

**FIGURE 5:**
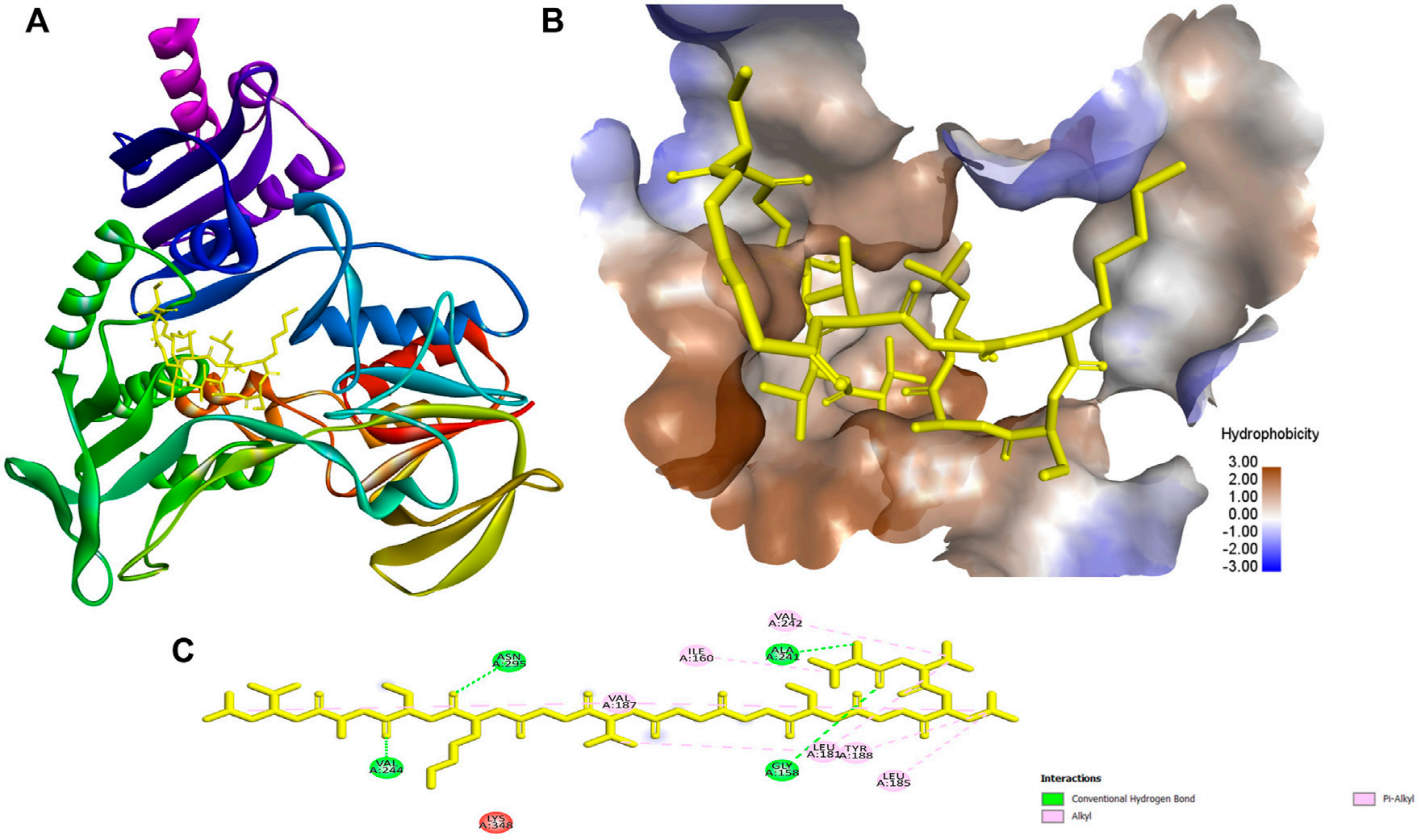
The docking results and the interaction of ACAP-V with NADH peroxidase (PDB Id: 1 nhp). **(A)** 3-D structure of NADH peroxidase-ACAP-V complex. **(B)** The 3-D hydrophobicity surface plot at the binding site and **(C)** 2D diagram of the NADH peroxidase-ACAP-V molecular interactions. The interactions with residues are shown in different colours. ACAP-V formed four polar with Val244, Asn295, Gly157, and Ala241. Also eight hydrophobic interactions between ACAP-V and Val187, Ile160, Val242, Leu181, Try188, and Leu181, Val187, and Leu185 of NADH peroxidase were demonstrated.

**FIGURE 6:**
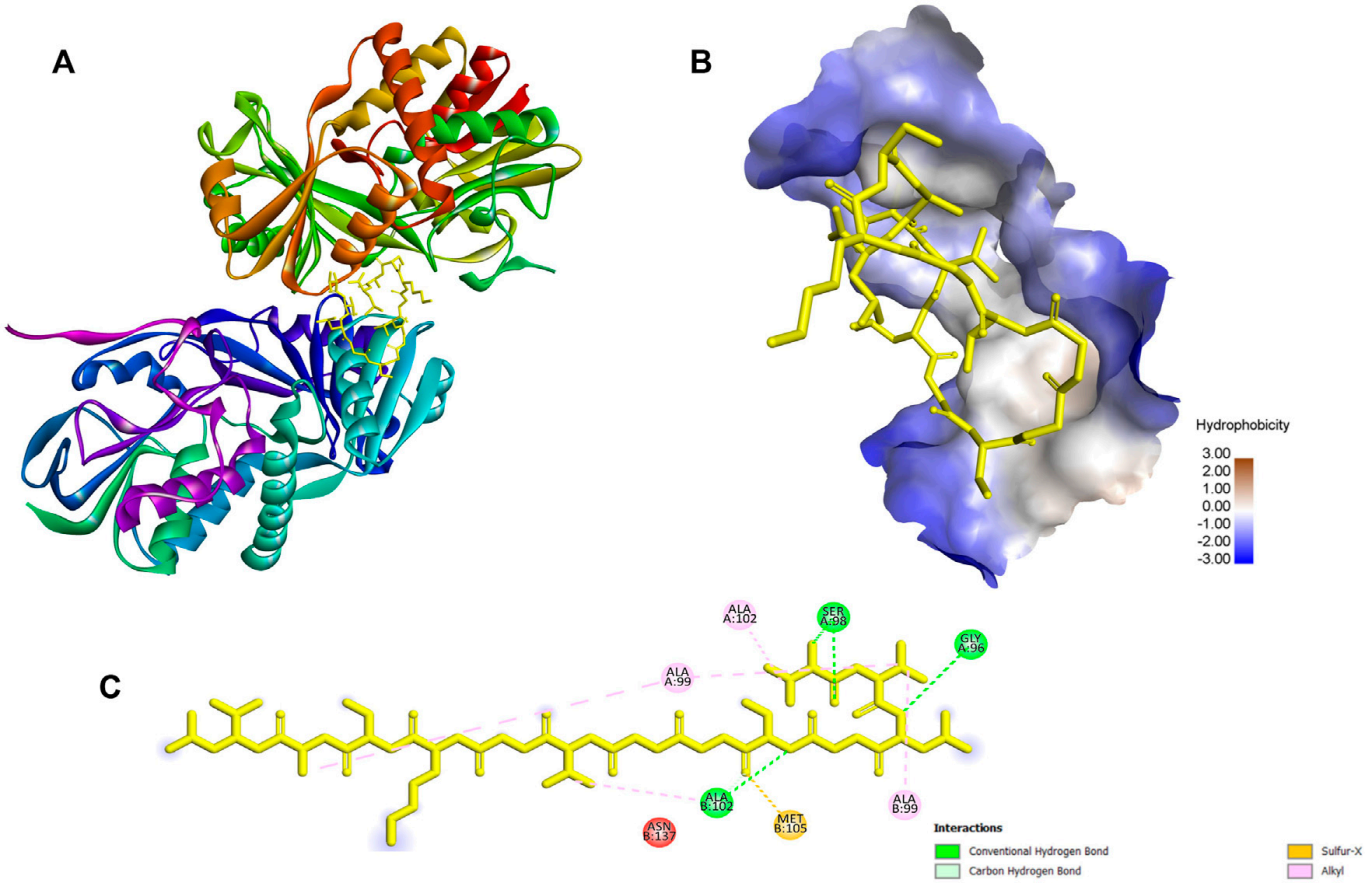
The docking results and the interaction of ACAP-V with against Monomeric sarcosine oxidase (PDB Id: 2gb0). **(A)** 3-D structure of Monomeric sarcosine oxidase-ACAP-V complex. **(B)** The 3-D hydrophobicity surface plot at the binding site. **(C)** 2D diagram of the Monomeric sarcosine oxidase-ACAP-V molecular interactions. The interactions with residues are shown in different colours. ACAP-V peptide formed four hydrogen bonds with Ser98, Gly96, Ser98 and Ala102; five alkyl bonds with Ala A99, Ala B99, Ala A99, Ala A102, and Ala B102 and a sulfer-x bond with Met105 of Monomeric sarcosine oxidase.

The highest binding energies for ACAP-V were observed against Monomeric sarcosine oxidase (PDB Id: 2gb0; −9.40 kcal/mol) whereas its highest global energy was observed against NADH peroxidase (PDB Id: 1 nhp; BE: −88.84 kcal/mol). The best docking pose of ACAP-V within the binding pockets of NADH peroxidase revealed four hydrogen bond interactions and up to eight hydrophobic bond interactions (Figure 5). On the other hand, Monomeric sarcosine oxidase demonstrated four hydrogen bonds, five hydrophobic bonds and a covalent bond interaction with ACAP-V (Figure 6).

**FIGURE 7:**
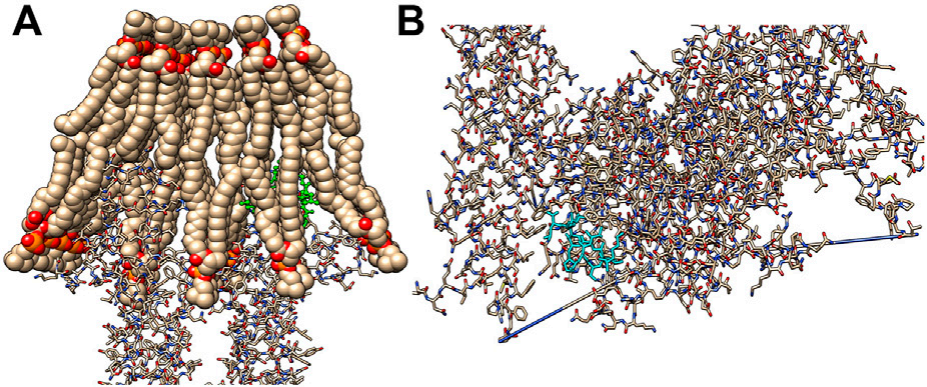
Interactions of the ACAPs with the bacterial cell membrane (PDB Id: 2w6d). **(A)** ACAP-V (yellow) bound to the outer pocket on Chain A of the bacterial cell membrane intrinsic protein in the phospholipid bilayer. **(B)** ACAP-IV (cyan) boundan to inner pocket on chain A of the bacterial cell membrane intrinsic protein. The images were rendered on the *Chimera 1.15*.

The top binding energy score poses demonstrated that both ACAPs could penetrate the bacterial cell membrane (Figure 7), with ACAP-V still yielding higher global energy (−87.35 kcal/mol) compared to ACAP-IV (−87.08 kcal/mol) and binding energy of −5.0 kcal/mol and 4.9 kcal/mol respectively. The two promising antimicrobial peptides (ACAP-IV and ACAP-V) varied in chemical structures, with peptide ACAP-IV demonstrating a longer alkyl chain length of the substituent group than that of ACAP-V. Besides, ACAP-IV had an arene group unlike ACAP-V. Much as both ACAPs had equal number of amides function groups, peptide ACAP-IV demonstrated more amines groups (04) than peptide ACAP-V (02). Such structural components of the ligands are instrumental in their interactions at the receptor binding site to cause an activity.

### *In vitro* antibacterial activity of ACAP-IV and ACAP-V

The antibacterial activity of the synthesized ACAP-IV and ACAP-V was evaluated. Contrary to the results of the docking exercises, ACAP-IV demonstrated a higher antibacterial activity compared to ACAP-V with the MIC of 520.7 ± 104.3 μg/ml and 1666.7 ± 416.7 μg/ml against *E. coli* and *S. aureus*, respectively (Table 6).

**TABLE 6:** Minimum inhibitory concentration of ACAPs on *E. coli* and *S. aureus*.

Run	MIC (µg/ml)	
	*E. coli*	*S. aureus*
ACAP-IV	520.7 ± 104.3	1666.7 ± 416.7
ACAP-V	625.0 ± 00	2083.3 ± 416.7
Ciprofloxacin	104.0 ± 26.0	130.7 ± 25.4
1X PBS	ND	ND

Data are expressed as Mean ± Standard Error of Mean; ND, not detected.

## Discussion

In the wake of the increasing resistance of pathogens to traditional antibiotics, AMPs are a potential alternative to traditional antibiotics. The primacy of the AMPs is the ability to directly target bacteria cell membrane (Kumar et al., 2018), hence reducing the likelihood of development of bacterial resistance. Besides, pathogens are unable to vary their negatively charged cell membrane composition to evade the electrostatic interactions with the cationic peptides (Brogden, 2005). However, developing new peptide therapies through the traditional screening process has been hampered by huge costs and the labour-intensive nature of basic research. On the other hand, machine learning approaches such as virtual screenings together with ADMET sieving can accelerate the screening of potentially safe and most promising active novel drug leads from huge libraries with high precision and efficiency while saving time and resources. These predictions can guide the selection of drug candidates before to cell and animal studies, significantly reducing development time (Lionta et al., 2014). The present study ably demonstrated this scenario by screening potentially safe and efficacious fish-derived AMPs previously identified from the skin mucus of African catfish.

The ADMET studies were conducted to evaluate the pharmacokinetic parameters (Dong et al., 2018) of the fish peptides, including absorption, distribution, metabolism, excretion, and toxicity properties. Human Intestinal Absorption (HIA) was evaluated as an important factor that affects bioavailability (Radchenko et al., 2016); both peptides (ACAP-IV and ACAP-V) were HIA+, indicating absorbability in the human intestine. Similar findings were reported for tri-peptides from rainbow trout fish (*Oncorhynchus mykiss*) (Yu et al., 2018) and angiotensin-converting enzyme inhibitory (ACEI) peptides (Fan et al., 2020). The Central Nervous System (CNS) permeability was determined by the blood-brain barrier (BBB) score. The BBB score depicts the capability of a molecule to penetrate the semipermeable blood-brain barrier that guards the CNS against potentially harmful substances. All the fish-derived antimicrobial peptides studied had a BBB score below the threshold (BBB score <0.1) unlike amyloid beta (Aβ) peptides (Tran et al., 2019) and chloroquine (Parashar et al., 2021). This finding implies the peptides are unable to cross the BBB, signifying potential safety to the CNS. However, BBB permeability is essential for treating infectious conditions of the CNS such as bacterial meningitis (Tunkel et al., 1991). Hence, seven AMPs are inferior drug candidates for the treatment of CNS infections but, the peptides could be modified structurally if needed.

Further ADMET predictions showed that none of the seven peptides inhibits the cytochrome P450 enzymes (CYP1A2 and CYP3A4), indicating their ability to allow drug metabolism as well as their potential clearance. Cytochrome P450 (CYP) system is a family of heme-containing enzymes with about 57 isoforms including CYP1A2, CYP2C9, CYP2C19, CYP2D6, and CYP3A4, among others. These CYP enzymes detoxify foreign materials and metabolize drugs in the liver (Zhou, 2008). Inhibition of CYP enzymes by the antimicrobial peptides induces hepatotoxicity (Han et al., 2018) due to the accumulation of reactive metabolites and other toxic substances (Fontana et al., 2005; Hakkola et al., 2020). Inhibition of the CYP enzymes by the antimicrobial peptides would cause potential impairment of drug clearance (Orellana and Guajardo, 2004). In contrast, the FAM1 cyclic peptides (Saragih et al., 2020) and polysaccharide peptides from *Coriolus versicolor* (Yeung and Or, 2012), were shown to inhibit the CYP1A and CYP3A4 enzymes. With regards to cardiotoxicity, all the screened peptides were predicted to be non-hERG blockers. The potassium flux in the myocardium is controlled by the by Human ether ago-go gene (hERG). The hERG blocking by a drug molecule can potentially lead to serious heart complications such as Long QT syndrome (LQTS) (Hoffmann and Warner, 2006). This syndrome affects the heart repolarization after a heartbeat due to improper potassium influx and can lead to a high risk of an irregular heartbeat manifested through fainting, drowning, seizures, or sudden death (Sanguinetti and Tristani-Firouzi, 2006). Similar findings have been reported for Vasoactive Proline-Rich Oligopeptide (hERGI) (Arcanjo et al., 2017), Eleutheroside (hERGI and hERGII) (Ahmed et al., 2020) among others, with no mutagenic, no carcinogenic, or drug-induced liver injury. Thus, all the studied ACAPs were predicted safe and promising, with ACAP-IV and ACAP-V demonstrating the best ADMET profiles. Much as, traditional therapeutics are known to be small (<500 Da), all the 7 ACAPs in the present study demonstrated molecular weight beyond 500 Da with the least being 1005.57 Da, thus, violating Lipinski’s Rule of 5 (Lipinski et al., 2012). As a rule of thumb, Lipinski’s Rule of five evaluates if a bioactive compound is likely to make it as an orally administered active drug in humans. Violation of this rule by the ACAPs in the present study indicates their potential poor absorption due to greater lipophilicity and lower water solubility, thereby reducing their bioavailability (Guimarães et al., 2012). Therefore, future developments of such ligands have to incorporate non-traditional drug delivery systems including nanocarriers (Li et al., 2012) and use of computational tools to identify as well as modify structural physiochemical parameters like hydrogen bond interactions, macrocyclization for their adequate accumulation at the target site (Pauletti et al., 1997; Hou et al., 2017).

The two antimicrobial peptides (ACAP-IV and ACAP-V) were docked against their respective target proteins. The molecular docking technique permits simultaneous investigation of thousands of molecules and later incorporates the ligand-target interactions to rank such molecules based on their binding affinities (Lionta et al., 2014; Kumar et al., 2020). The technique utilizes machine learning search algorithms to predict ligand-target binding sites and affinity (Shukla et al., 2020) and has been widely used to aid the discovery of several drugs including Captopril, Dorzolamide, Saquinavir, Zanamivir, Oseltamivir, Aliskiren, Boceprevir, Nolatrexed, TMI-005, LY-517717, Rupintrivir and NVP-AUY922 (Talele et al., 2010). Additionally, novel drug leads such as anticancer phytochemicals, Miyakamide B2 and Iryantherin D (Kotadiya and John, 2015); antimicrobial peptides, A15_B and A15_E (Okella et al., 2020); Canola proteins, napin and cruciferin (Rahman et al., 2020); plant-isolated anti-tubercular (9z, 12z)-Octadeca-9,12-dienoic acid (Mtewa et al., 2021), were uncovered through molecular docking. The docking results showed ACAP-V had the strongest binding affinities with a binding energy of −9.40 kcal/mol and global energy of −88.84 kcal/mol against monomeric sarcosine oxidase (PDB Id: 2gb0) and NADH peroxidase (PDB Id: 1 nhp), respectively. The best docking pose revealed that ACAP-V formed four polar and eight hydrophobic interactions with NADH peroxidase (PDB Id: 1 nhp); the Val244, Asn295, Gly158, and Ala241 of NADH peroxidase formed hydrogen bonds with ACAP-V residues, whileVal187, Ile160, Val242, Leu181, Try188 Val187, Leu181, and Leu185 formed hydrophobic interactions with ACAP-V (Alkyl and Pi-alkyl).

Such interactions depend on the structure of the ligands and are crucial for the binding of the drug to the target, physiological changes during the binding (bioactivity), as well as drug elimination. Aromantic rings are for example vital in fostering both the π-π and hydrogen bond interactions. Steric bulk too, enhances hydrogen bond interactions. The amines and amides functional groups on the other hand, are responsible for dipole-dipole as well as additional hydrogen interactions between the ligands and the target protein (Ertl et al., 2020). Although hydrophobic interactions are much weaker, they allow momentary interactions and unlike the covalent bonds that are usually hard to break, they easily break to release the ligands after their interactions with the target protein to enhance drug excretion (Varma et al., 2010). The hydrogen bonds on the other hand, enhances optimal interactions that allows timely dislodgment of the drug molecule from the binding site and hence, excretion after effecting action (Varma et al., 2010; Chen et al., 2016). Therefore, the four hydrogen bonds in the present study were the most essential given that, NADH peroxidase is a critical enzyme in bacterial tolerance mechanism. In this mechanism, NADH peroxidase catalyzes the degradation/inactivation of hydrogen peroxide produced within the cell by metabolic pathways, such as glycerol metabolism and dismutation of superoxide, before it damages essential cellular components (Miyoshi et al., 2003; Naraki et al., 2020). Inhibition of such enzymes by ACAP-V can potentially lead to the accumulation of cell-generated hydrogen peroxide leading to bacterial cell distraction and death.

Conversely, monomeric sarcosine oxidase catalyzes the oxidative demethylation of sarcosine (N-methlyglycine) to form glycine and formaldehyde. The enzyme has a co-factor (flavin) both covalently and non-covalently bound to it in a 1:1 M ratio (Trickey et al., 1999). Sarcosine is the sole source of energy for many microorganisms capable of expressing sarcosine oxidase. Molecular docking revealed that the ACAP-V peptide formed four hydrogen bonds with Ser98, Gly96, Ser98 and Ala102; five alkyl bonds with Ala A:99, Ala B:99, Ala A:99, Ala A:102 and Ala B: 102 and a sulfer-x bond with Met105 of monomeric sarcosine oxidase. These interactions inhibit the demethylation of sarcosine, thereby depriving energy production in such organisms. This can potentially lead to the death of microbes whose sole source of energy is sacrosine (Hassan-Abdallah et al., 2006; Zhao et al., 2008).

Similarly, the action mechanism for cell lysis revealed that both ACAP-V and ACAP-IV interact with chain A of bacterial cell membrane extrinsic protein (PDB Id: 2dw6). Related findings were reported for cruzioseptin peptides (Cuesta et al., 2021) and flocculating polypeptides (Garcia et al., 2019). This bacterial cell membrane enzyme (PDB Id: 2dw6) is vital in the synthesis of unsaturated fatty acids, a key component of the phospholipid bilayer (Kopp et al., 2020), through a series of dehydration and isomerization reactions. Inhibition of the enzyme thus impedes the cell membrane biosynthesis process (Leesong et al., 1996). Moreover, such interactions between the AMPs and bacterial cell membrane can potentially aid both cell membrane penetration (Lohner and Prossnigg, 2009) and “detergent-like” distraction of the bacterial cell membrane (Oren and Shai, 1998), leading to cell death (Okella et al., 2022).

Contrary to the docking exercises, *in vitro* validations revealed that ACAP-IV (KVSKVLHKAIL) demonstrated a higher *in vitro* antimicrobial activity than ACAP-V (VVLGSGGVGKSAL). However, its MIC of 520.7 ± 104.3 and 1666.7 ± 416.7 on *E. coli* and *S. aureus* respectively indicate a lower antibacterial activity when compared to Pelteobagrin (GKLNLFLSRLEILKLFVGAL) from yellow catfish, *Pelteobagrus fulvidraco* (MIC, 2–64 μg/ml) (Su, 2011); Gs D (FIGGIISFFKRLF) from Goldenstriped soapfish, *Grammistes sexlineatus* (MIC, 13.3–50 μg/ml) (Sugiyama et al., 2005); Misgurin (RQRVEELSKKGAAARRRK) from Pond Loach, *Misgurnus anguillicaudatus* (MIC, 4–16 μg/ml) (Park et al., 1997). During a solid-phase peptide synthesis, a condensation reaction between the carboxyl-terminus and amino-terminus occurs to link amino acids through amide/peptide bonds (Marglin and Merrifield, 1970). Variation in reaction conditions as well as various side chain protecting strategies during the chemical synthesis process impact the bioactivity of the synthesized products (Marglin and Merrifield, 1970; Abdel-Aal et al., 2020). Further, Kęska and Stadnik (2017) maintain that amino acid composition, structure, and physicochemical properties account for the wide variations in the antimicrobial activity of different peptides. These could explain the variation. However, the wide variations can be reduced by ligand optimization.

## Conclusion

The present study revealed the ADMET profiles and mode of action of the previously identified African catfish antimicrobial peptides. Peptide ACAP-IV and ACAP-V demonstrated outstanding ADMET profiles with, ACAP-IV emerging as a more promising antimicrobial peptide at *in vitro* level. Such antimicrobial candidates can potentially serve as lead molecules with potential application in the pharmaceutical, nutraceutical and cosmetic industries. However, we recommend primary structure optimization and heterologous expression of ACAP-IV, ACAP-V and their analogous to enhance their *in vitro* antibacterial activity to less than 100 μg/ml.

## Data Availability

The original contributions presented in the study are included in the article/Supplementary Material, further inquiries can be directed to the corresponding author.
