# Supplementary figures and images for: ADMET profiling and molecular docking of potential antimicrobial peptides previously isolated from African catfish, Clarias gariepinus
Source: Front Mol Biosci. 2022 Dec 8;9:1039286. doi: 10.3389/fmolb.2022.1039286 (PMC9772024; doi:10.3389/fmolb.2022.1039286)

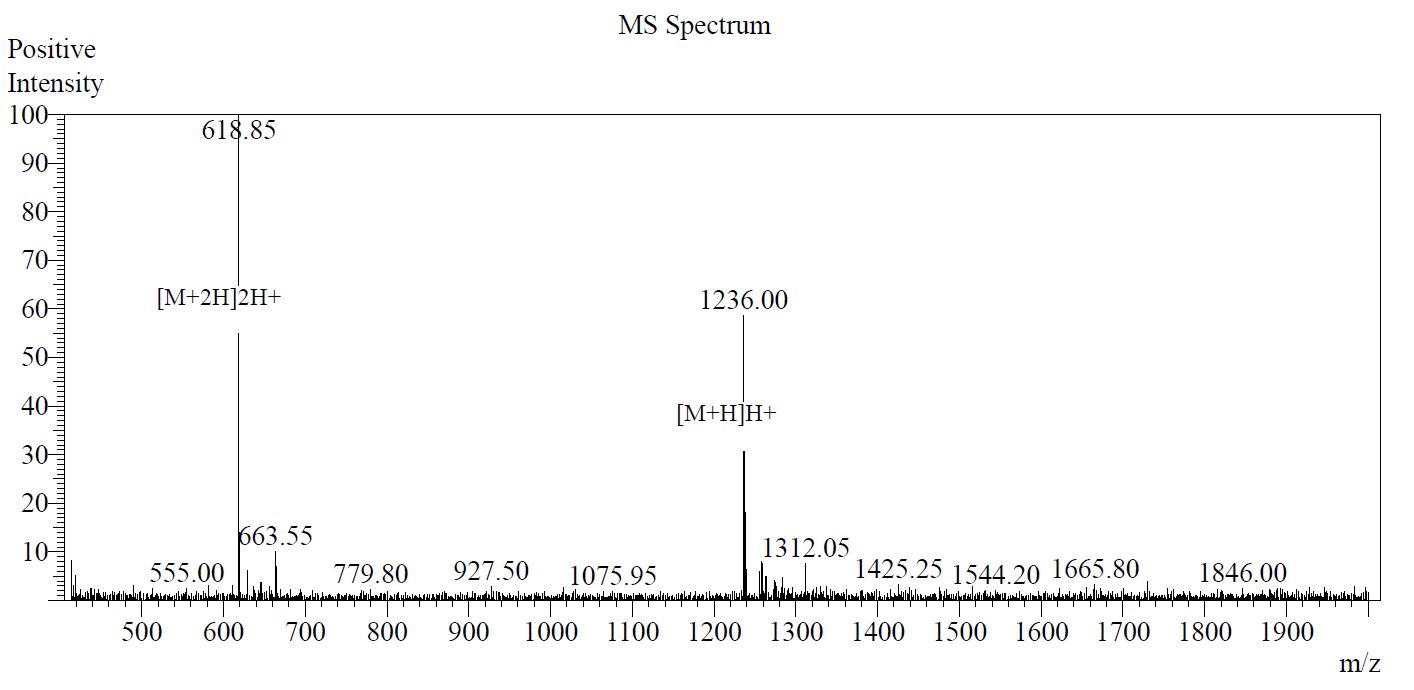

Supplement: Supplementary file 1 [file Image3.JPEG]

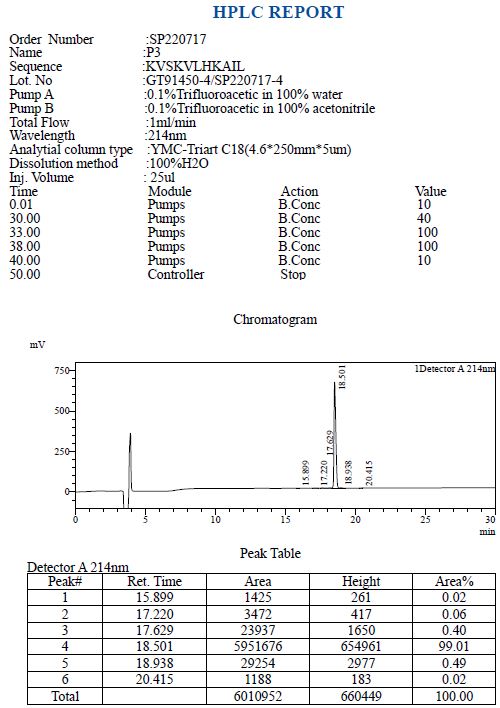

Supplement: Supplementary file 3 [file Image1.JPEG]

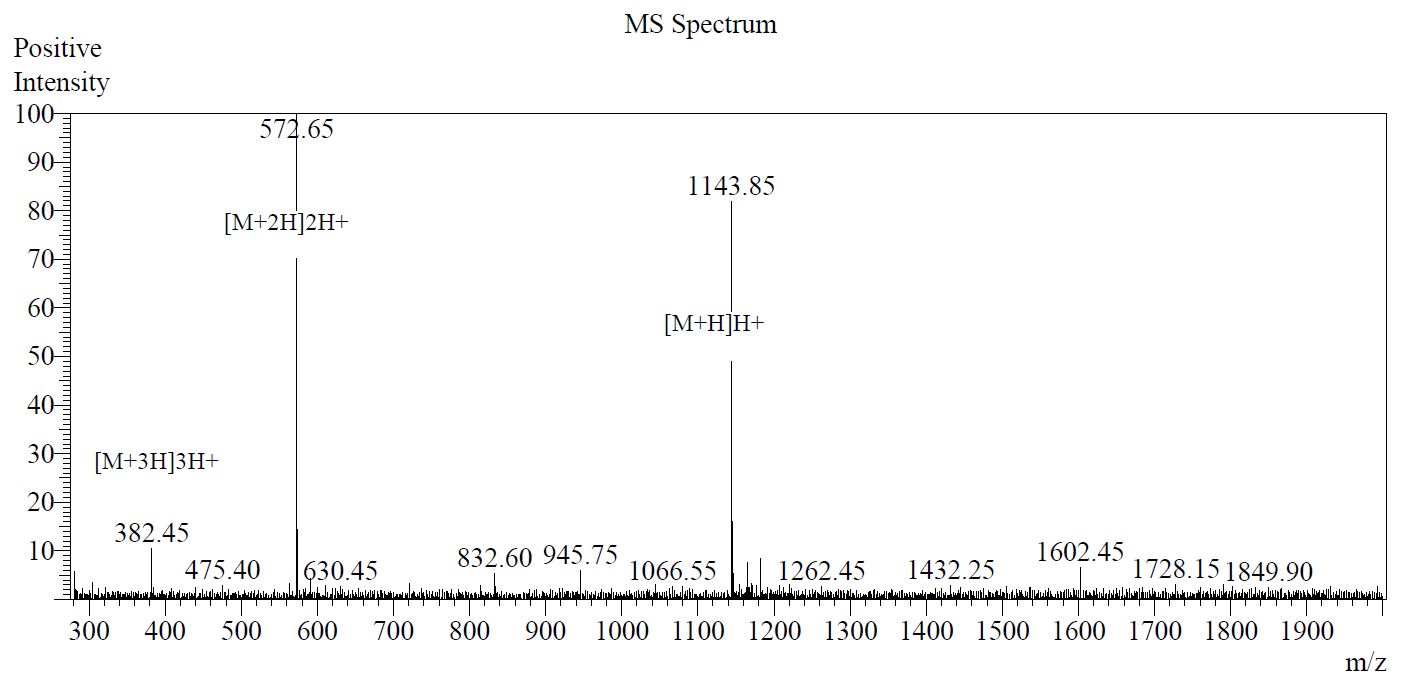

Supplement: Supplementary file 4 [file Image4.JPEG]

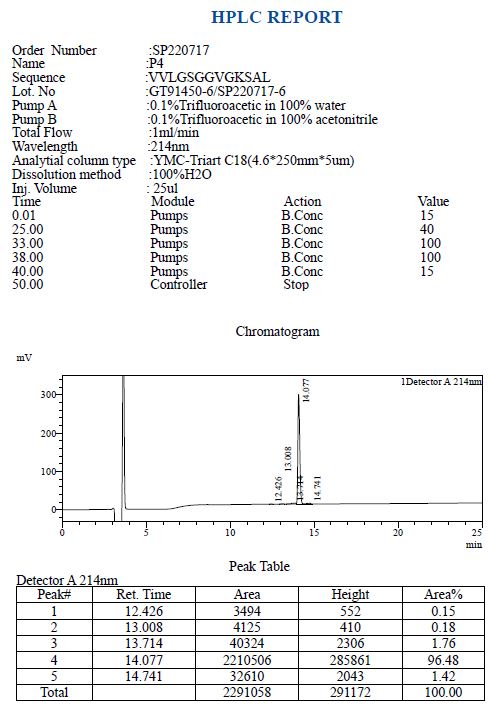

Supplement: Supplementary file 5 [file Image2.JPEG]
