# Supplementary material for: ADMET profiling and molecular docking of potential antimicrobial peptides previously isolated from African catfish, Clarias gariepinus
Source: Front Mol Biosci. 2022 Dec 8;9:1039286. doi: 10.3389/fmolb.2022.1039286 (PMC9772024; doi:10.3389/fmolb.2022.1039286)
Supplement: Supplementary file 2 [file Table1.docx]

| **Supplementary Table S1: Templates used in I-TASSER 3D structure prediction of ACAPs** | | | | | | | | |  |
| --- | --- | --- | --- | --- | --- | --- | --- | --- | --- |
|  |  | **ACAP-IV** | |  |  |  | **ACAP-V** |  |  |
| **PDB Hit** | **Iden1** | **Iden2** | **Cov** | **Norm. z-score** | **PDB Hit** | **Iden1** | **Iden2** | **Cov** | **Norm. z-score** |
| 4a1gE | 0.27 | 0.27 | 1.00 | 2.21 | 3oesA | 0.54 | 0.54 | 1.00 | 1.29 |
| 4yuuW | 0.36 | 0.36 | 1.00 | 1.04 | 5lqwX | 0.33 | 0.46 | 0.92 | 1.22 |
| 4yuuW | 0.36 | 0.36 | 1.00 | 1.36 | 502rZ | 0.15 | 0.15 | 1.00 | 1.31 |
| 3w3wB | 0.50 | 0.46 | 0.91 | 2.10 | 5f3yB | 0.23 | 0.23 | 1.00 | 2.94 |
| 5o5jB | 0.36 | 0.36 | 1.00 | 1.01 | 3c5cB | 0.62 | 0.61 | 1.00 | 1.27 |
| 5o5jB | 0.36 | 0.36 | 1.00 | 1.14 | 6f0qA | 0.31 | 0.31 | 1.00 | 1.07 |
| 4irvE | 0.18 | 0.18 | 1.00 | 1.89 | 5w4kA | 0.10 | 0.15 | 0.77 | 1.00 |
| 3s0rA | 0.36 | 0.36 | 1.00 | 1.10 | 7b5mP | 0.22 | 0.15 | 0.69 | 2.12 |
| 3ffdP | 0.27 | 0.27 | 1.00 | 1.74 | 3cbqA | 0.54 | 0.54 | 1.00 | 1.27 |
| 1xc0A | 0.36 | 0.36 | 1.00 | 1.09 | 1ml8A | 0.08 | 0.31 | 1.00 | 1.06 |
