# Supplementary material for: ADMET profiling and molecular docking of potential antimicrobial peptides previously isolated from African catfish, Clarias gariepinus
Source: Front Mol Biosci. 2022 Dec 8;9:1039286. doi: 10.3389/fmolb.2022.1039286 (PMC9772024; doi:10.3389/fmolb.2022.1039286)
Supplement: Supplementary file 6 [file Table2.doc]

**Supplementary Table S2: Target proteins utilized in the docking exercise**

| **Ligands** | **Target Protein name** | **Classification** | **PDB Id** | **Identity (%)** | **z-score** | **Organism** |
| --- | --- | --- | --- | --- | --- | --- |
| ACAP-IV | Ferredoxin-dependent glutamate synthase 2 | Lyase | 1ofd | 60.0 | 96.8 | *Synechocystis sp* |
|  | Penicillin-binding protein 2x | Biosynthesis protein | 1mwt | 62.5 | 85.1 | *Streptococcus pneumoniae* |
|  | 30S ribosomal protein S13 | RNA binding proteins | 4v6l | 60.0 | 81.1 | *Escherichia coli* |
|  | Aminoglycoside N(6')-acetyltransferase type 1 | Transferase | 1s3z | 33.3 | 79.1 | *Salmonella enteritidis* |
|  | Acyl-homoserine lactone acylase PvdQ | Hydrolyse inhibitor | 4wks | 62.5 | 78.8 | *Pseudomonas aeruginosa* |
| ACAP-V | Flavohemoprotein | Lipid binding protein | 3ozw | 53.8 | 89.1 | *Cupriavidus necator* |
|  | Thioredoxin reductase | Oxidoreductase | 1f6m | 50.0 | 84.4 | *Escherichia coli* |
|  | |  | | --- |   NADH peroxidase | Oxidoreductase | 1nhp | 58.3 | 84.1 | *Enterococcus faecalis* |
|  | Fumarate reductase flavoprotein | Oxidoreductase | 1kf6 | 50.0 | 79.5 | *Escherichia coli* |
|  | Monomeric sarcosine oxidase | Oxidoreductase | 2gb0 | 41.7 | 79.0 | *Bacillus sp.* |
|  | Dihydrolipoyl dehydrogenase | Oxidoreductase | 1ojt | 66.7 | 77.5 | *Neisseria meningitidis* |
